# Validation of automated pipeline for the assessment of a motor speech disorder in amyotrophic lateral sclerosis (ALS)

**DOI:** 10.1177/20552076231219102

**Published:** 2023-12-21

**Authors:** Leif ER Simmatis, Jessica Robin, Timothy Pommée, Scotia McKinlay, Rupinder Sran, Niyousha Taati, Justin Truong, Bharatkumar Koyani, Yana Yunusova

**Affiliations:** 1 Department of Speech-Language Pathology, Temerty Faculty of Medicine, 7938 University of Toronto, Toronto, ON, Canada; 2 KITE-Toronto Rehabilitation Institute, 7989 University Health Network, Toronto, ON, Canada; 3 Winterlight Labs Inc., Toronto, ON, Canada; 4 282299 Sunnybrook Research Institute, Sunnybrook Health Sciences Centre, Toronto, ON, Canada; 5 7845 Regeneron Pharmaceuticals Inc., Tarrytown, NY, USA

**Keywords:** Motor speech, speech, amyotrophic lateral sclerosis, validation, digital biomarkers

## Abstract

**Background and objective:**

Amyotrophic lateral sclerosis (ALS) frequently causes speech impairments, which can be valuable early indicators of decline. Automated acoustic assessment of speech in ALS is attractive, and there is a pressing need to validate such tools in line with best practices, including analytical and clinical validation. We hypothesized that data analysis using a novel speech assessment pipeline would correspond strongly to analyses performed using lab-standard practices and that acoustic features from the novel pipeline would correspond to clinical outcomes of interest in ALS.

**Methods:**

We analyzed data from three standard speech assessment tasks (i.e., vowel phonation, passage reading, and diadochokinesis) in 122 ALS patients. Data were analyzed automatically using a pipeline developed by Winterlight Labs, which yielded 53 acoustic features. First, for analytical validation, data were analyzed using a lab-standard analysis pipeline for comparison. This was followed by univariate analysis (Spearman correlations between individual features in Winterlight and in-lab datasets) and multivariate analysis (sparse canonical correlation analysis (SCCA)). Subsequently, clinical validation was performed. This included univariate analysis (Spearman correlation between automated acoustic features and clinical measures) and multivariate analysis (interpretable autoencoder-based dimensionality reduction).

**Results:**

Analytical validity was demonstrated by substantial univariate correlations (Spearman's *ρ* > 0.70) between corresponding pairs of features from automated and lab-based datasets, as well as interpretable SCCA feature groups. Clinical validity was supported by strong univariate correlations between automated features and clinical measures (Spearman's *ρ* > 0.70), as well as associations between multivariate outputs and clinical measures.

**Conclusion:**

This novel, automated speech assessment feature set demonstrates substantial promise as a valid tool for analyzing impaired speech in ALS patients and for the further development of these technologies.

## Introduction

Amyotrophic lateral sclerosis (ALS) is an incurable and progressive motor neuron disease with an incidence of approximately 2/100,000 individuals per year in Canada.^1^ Bulbar motor symptoms affecting speech and swallowing functions present at disease onset in approximately 30% of patients^2^; they also develop with disease progression in over 80% of patients with spinal onset ALS.^3,4^ Up to 50% of patients with ALS demonstrate cognitive impairments (including cognitive–linguistic deficits), and approximately 10% meet criteria for frontotemporal dementia.^5,6^ Both motor and cognitive linguistic abnormalities affect speech output in this disease. The pervasiveness of these impairments in ALS has made speech a valuable disease marker, and there has been a great effort to develop speech-based assessment tools for people with ALS.

Since the 1980s, laboratory studies of speech have been focused on the identification of acoustic features that correlate with the presence, severity, and progression of dysarthria in ALS.^7–12^ This historical work led to the growing interest for identification of acoustic biomarkers in ALS. In turn, it also led to the development of several home-based assessment technologies that utilize consumer-grade audio recording equipment and analytic pipelines. Examples have included Modality.AI,^13^ Beiwe,^14,15^ and Aural Analytics.^16^ These technologies have demonstrated the potential of automated assessment of speech disorders and have opened the door for further research on novel platforms for detecting and quantifying the severity of ALS. Another app for speech assessment has been developed by Winterlight, which combines both cognitive–linguistic (e.g., lexical diversity) and acoustic features (e.g., mean fundamental frequency). To contextualize Winterlight's role in digital health more broadly, their assessment system was designed for the use with tablets and smartphones in trial settings, although the recording app can function separately from the automated analysis pipeline. This pipeline has been validated in the context of detection and tracking of cognitive–linguistic deficits in a variety of neurodegenerative and psychiatric diseases.^17–21^ However, Winterlight's diverse acoustic feature set has not been validated for the assessment of *motor* speech disorders (i.e., dysarthria) that are common and in many cases might coexist with cognitive–linguistic deficits, such as in ALS.

Therefore, in the present study, we sought to validate Winterlight's acoustic feature set in a large cohort of individuals with ALS that presented with dysarthria of varying severity and characteristics. We set out to follow the best practices for development of digital biomarkers established via the verification, analytical validation, and clinical validation (V3) framework^22,23^ and by the Food and Drug Administration (FDA)'s Biomarkers, EndpointS, and other Tools (BEST) guidelines.^24^ Objective 1 was to perform an analytical validation of the Winterlight pipeline, in which we compared Winterlight's acoustic feature set with similar features estimated using lab-standard software and processes. Objective 2 was to perform clinical validation, which involved comparing Winterlight's features to a series of clinical speech/overall function measures that have been established in ALS research and clinical trials. We formulated two hypotheses (1) that Winterlight's features would correspond strongly to salient lab-based features and (2) that Winterlight's features would be able to capture relevant disease-related phenomena in clinical subgroups.

## Methods

### Participants and clinical characteristics

This study had an observational (cohort) retrospective cross-sectional study design. Participants were recruited previously as part of longitudinal studies on tracking bulbar dysfunction in patients with ALS between 2008 and 2019. Inclusion criteria were a diagnosis of ALS and fluency in English. Exclusion criteria were signs of cognitive impairment (Montreal cognitive assessment (MoCA) score of <26/30) and history of other neurological diseases or speech disorders. For this analysis, participants were selected based on the presence and quality of the data. In this sample, we included 122 participants who provided recordings over the course of their disease progression. Their demographic characteristics are shown in Table 1. Only the final recording session from each individual was analyzed in the present study to capture a greater range of dysarthria severity. Informed consent prior to participation was collected in accordance with the Declaration of Helsinki.

**Table 1.** Demographic and clinical summary of the ALS cohort (at analyzed timepoint).

Demographic/measure	Value	Missing values (*N*)	Subgroup sizes Bamboo; Phonation; DDK
*N* (% female)	122 (39%)	-	-
Years since onset	2.8 [2.7]	-	-
Bulbar onset (*n*, % of total)	13 (11%)	2	(13/106); (12/85); (9/76)
Age	59 [14]	28	-
ALSFRS-R total	35 [12]	28	(46/47); (38/40); (34/36)
ALSFRS-R bulbar	11 [3]	28	(44/49); (39/39); (34/36)
ALSFRS-R respiratory	10 [6]	32	(31/58); (28/48); (29/39)
SIT-rate (WPM)	148 [51]	4	(70/47); (51/43); (45/40)
SIT-intelligibility (%)	99 [5]	4	(31/86); (23/71); (17/68)
FVC (%)	75 [30]	53	(37/31); (28/21); (25/20)

All summaries are either *n* (%) or *median* [*interquartile range (IQR)*]. ALSFRS-R = ALS Functional Rating Scale-Revised. SIT = Speech Intelligibility Test. FVC = forced vital capacity. WPM = words per minute. Subgroup sizes are shown as (more-severe/less-severe) for Bamboo, Phonation, DDK datasets (see Statistical analyses section).

The clinical characteristics of the sample are presented in Table 1. A series of standard clinical measures were used to describe the severity of the overall disease and dysarthria associated with ALS. The ALS Functional Rating Scale-Revised (ALSFRS-R)^25^ was used to evaluate overall function, as well as broad bulbar and respiratory functionality. The ALSFRS-R consists of 12 items that each correspond to an aspect of daily life functioning. These items are rated from 0 (unable to do) to 4 (normal level of function). The total score (FRS-total) therefore ranges from 0 to 48. The ALSFRS-R is further divided into subscales that measure specific components of overall function. Two that are relevant for the present work are the respiratory subscale (FRS-resp) and the bulbar subscale (FRS-bulb), each of which consist of three items and have a score range of 0–12.

The Speech Intelligibility Test (SIT) was used to assess the severity of dysarthria and consists of randomized sentences that provide measures of speech intelligibility and speaking rate.^26,27^ Participants read sentences of varying lengths, and a human rater transcribed these sentences and identified sentence boundaries. The SIT software estimated the percent of intelligible words based on broad transcription (SIT-I) as well as calculated the speaking rate (SIT-R) in words per minute (WPM). Finally, %forced vital capacity (%FVC) was used to estimate respiratory capacity of patients in this study. Briefly, %FVC is the maximal amount of air that can be exhaled following a full inhalation. It was recorded using spirometry.

### Tasks

Participants performed three speech tasks commonly used for the assessment of dysarthria. They include passage reading, vowel phonation, and diadochokinesis (DDK). Participants read the Bamboo Passage (“Bamboo”), which is 99 words in length and assesses various aspects of articulatory and respiratory motor function.^28^ Vowel phonation (“Phonation”) consisted of participants sustaining the vowel /a/ for as long as they comfortably could on a single breath. Finally, the DDK task consisted of participants repeating the syllable /ta/ as quickly and for as long as they comfortably could on a single breath (“DDK”). Data were recorded in a speech laboratory embedded into a multidisciplinary ALS clinic and conducted in a typical clinic room. The recordings were conducted using a high-quality digital recorder at 44.1 kHz in 16-bit resolution using a cardioid lavalier microphone. All recordings were analyzed in parallel using our in-lab methods and Winterlight's method, to ensure maximal comparability of feature outputs.

### Preprocessing of acoustic recordings

To prepare data for downstream analyses, preprocessing was performed on the raw acoustic samples. Noise reduction was performed using Praat.^29^ A minimum of 0.25 seconds (i.e., a minimum of ∼10,000 samples) was used for the spectral subtraction noise reduction algorithm,^30^ with a window length of 0.025 seconds. This follows the recommendations on the Praat noise reduction; we selected a total sample length that was at least several times the length of the window (https://www.fon.hum.uva.nl/praat/manual/Sound__Remove_noise___.html, retrieved 24 Nov 2022). Other settings for noise reduction included suppression range of 80 Hz to 10 kHz and 40 Hz smoothing.

Further semi-automated analysis of data quality was performed to ensure high-quality data was analyzed after noise reduction was performed. Thresholds were signal-to-noise ratio (SNR) of >30Db,^31^ clipping in fewer than 1% of data samples,^32^ and no unusual patterns of noise as evident by visual inspection of spectrograms. These steps were performed by a trained and experienced research assistant (JT), with uncertain cases resolved among JT, an experienced acoustic data researcher/speech–language pathologist (TP), and the first author (LERS).

### In-lab acoustic feature extraction

Following preprocessing, data were subjected to feature extraction using lab-standard methods and software. This feature extraction was conducted using manual and semi-manual analysis by highly trained research assistants.

For Bamboo, data were analyzed using speech and pause analysis (SPA) software^33^ as well as Parselmouth,^34^ the latter of which was used to create custom Python scripts. These features encompassed various aspects of the speech sample such as intensity/amplitudes, speech/pause segment durations, jitter/shimmer, etc. A total of 80 features were extracted from the Bamboo data.

For Phonation, Multi-Dimensional Voice Program (MDVP) and Assessment of Dysphonia in Speech and Voice (ADSV) software was used (both from KayPENTAX). Features encompassed cepstral peak prominence, jitter/shimmer, etc. Segments of 2–4 seconds in duration were selected for analysis, avoiding sections with excessive clipping (typically at the beginning of each sample). A total of 43 features were extracted from Phonation data.

Finally, DDK samples were analyzed using a recently validated MATLAB-based pipeline that extracted two features: rate (syllables per second) and cycle-to-cycle variability (CTV, i.e., the variability in inter-syllable timing).^35^

### Winterlight acoustic feature extraction

The Winterlight pipeline generated 775 features; of these, 555 are considered to be “text” or “transcript” based (i.e., linguistic) and 220 measures considered to be “acoustic.” The acoustic features include measures of F0, jitter, shimmer, intensity, and harmonic/noise ratio (HNR). Only acoustic features were evaluated here because the focus of this project was on validating Winterlight's acoustic features. We added three features that were semi-automatically extracted from the “transcript” category, specifically “speech rate,” “articulation rate,” and “average word duration” (i.e., measures of timing that correspond to our in-lab analyses). These features were automatically extracted from the manually generated transcripts of each sample and are also typically counted in the linguistic feature category. We removed mel-frequency cepstral coefficients (MFCCs) from the feature set, as we chose to focus on classical acoustic features in this analysis. In total, this left 53 features remaining in the dataset. The same feature set was extracted for each of the three tasks. See Supplementary Table 1 for a detailed summary of all extracted features per task for Bamboo, Phonation, and DDK using Winterlight's pipeline.

Winterlight's feature extraction approach consists of various manual and automated steps, which were performed by the company's transcription and processing teams. We provided speech samples to Winterlight that had been preprocessed following the above criteria, in order to ensure maximal comparability of the resulting features between lab-based and Winterlight analyses. Each sample was subject to initial quality check by Winterlight, followed by manual transcription of words/syllables (DDK only) to derive subsequent features.

### Statistical analyses

*Analytical validation (univariate and multivariate analyses)*: To subserve Objective 1 of determining the analytical validity of Winterlight's acoustic features, we compared them directly to lab-based acoustic features. This was done first using Spearman correlations between individual features from each dataset and then performing multivariate analyses in order to establish the high-level similarity between Winterlight and lab-based datasets. Correlations were deemed “very strong” when *ρ* > |0.90|, “strong” when |0.90| > *ρ* > |0.70|, and “moderate” when |0.70| > *ρ* > |0.50|.^36^ Multivariate analysis was accomplished using sparse canonical correlation analysis (SCCA), using the penalized matrix decomposition (PMD) method,^37^ and extracting two latent dimensions. SCCA was implemented in the Python “CCA-Zoo” package.^38^ Loadings for each dataset were extracted and compared using biplots. This enabled identifying features that covaried similarly across each dataset. Thresholds for substantial loadings were set at >|0.50|, which is a conservative cutoff based on thresholds used in exploratory factor analysis.^39^ Descriptive analysis was performed by inspecting biplots of component loadings to identify salient groups of features. All analytical validations were performed per task.

*Clinical validation (univariate and multivariate analyses):* For Objective 2 of clinically validating Winterlight's assessment system in ALS, we first performed univariate Spearman correlations between Winterlight's acoustic feature sets and clinical measures (e.g., FRS-bulb). Interpretation thresholds for the correlations were as above. These were repeated for data from each task separately.

Multivariate analysis was performed using a variational autoencoder (VAE)-based method called BasisVAE,^40^ which can extract interpretable feature groups. We first grouped participants based on their clinical scores or demographics and then trained BasisVAE. BasisVAE generated three outputs, which were subsequently analyzed using four downstream methods: step 1, low-dimensional representations of data (“embeddings”); step 2, feature groups; step 3, feature-level predictions; and step 4, clusters of correlations between feature-level predictions and clinical measures. These outputs were further analyzed to explore relationships between Winterlight's features and clinical measures.

We grouped the ALS cohort into “less-severe” and “more-severe” subgroups for six clinical measures of interest. ALSFRS-R data led to groupings using median values as thresholds: <35/48 (total score), <10/12 (FRS-resp), and <11/12 (FRS-bulb). SIT data led to groupings using established thresholds: <160 WPM for SIT-rate^41^ and 96% for SIT-intelligibility.^42^ %FVC data were used to group at an established threshold of <80%.^43^

In addition to the clinical subgroups, we considered the demographic subgroups of male and female participants. This is because we used primarily acoustic features, some of which are profoundly impacted by sex (e.g., fundamental frequency). Including sex subgroups enabled us to determine whether features that were strongly related to sex were also strongly related to other clinical measures (implying the potential for a confounding effect) or whether there were features that were specific to clinical measurements. Sex was numerically encoded to correspond to the approach taken for clinical measures (male = 1, female = 2).

**Figure 1.**
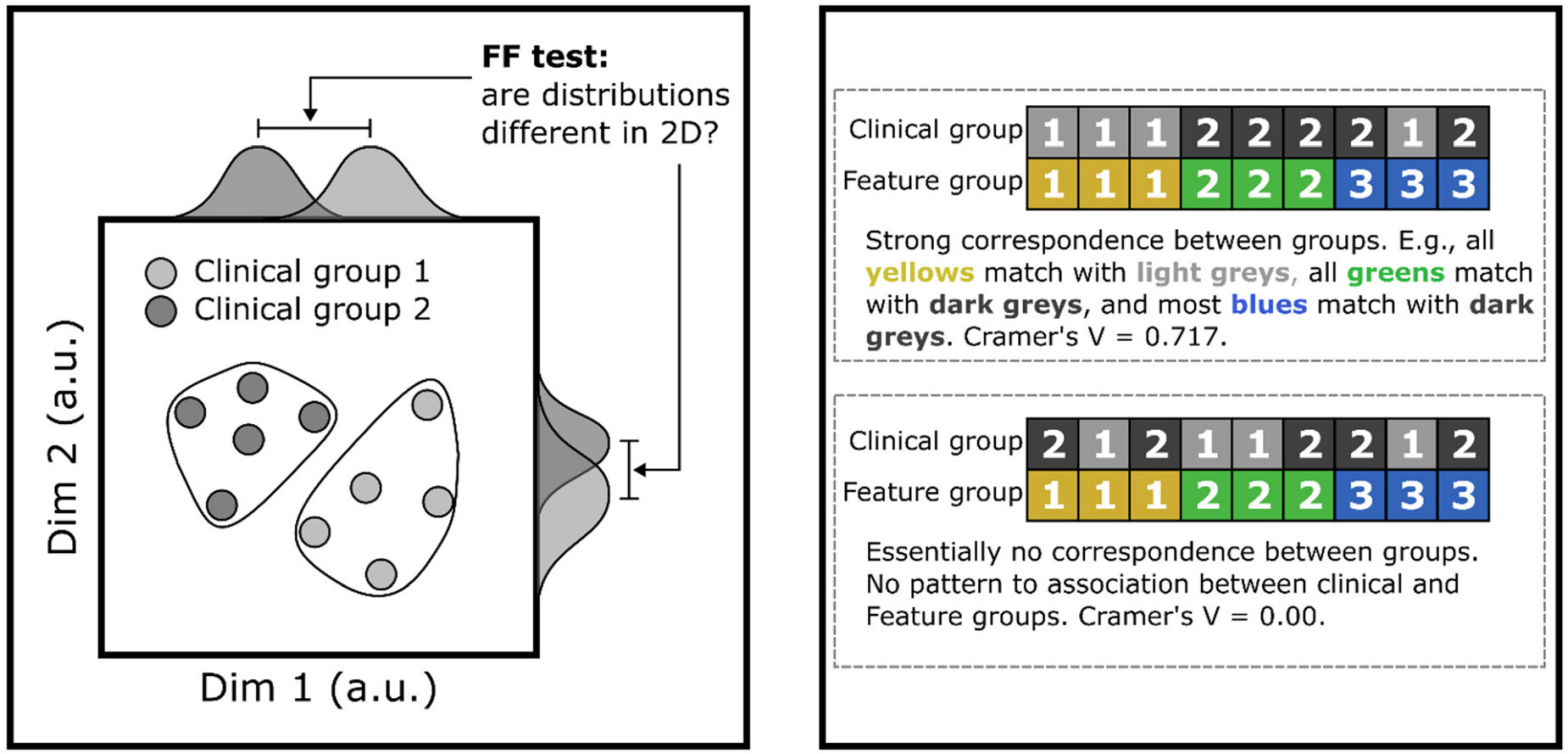
Examples (simulated data) of analyses using BasisVAE outputs. **Left panel**: Statistically comparing clinical subgroups using the Fasano–Franceschini (FF) test. Each circle is an embedding of an individual's high dimensional data in 2D space, color-coded by their clinical group. Marginal distributions (dark gray and light gray Gaussian shapes on top and right plot spines) are each partially separable; however it is clear from the 2D points that the 2D distributions are completely separable. The FF test captures this 2D difference. Note: “a.u.” = arbitrary units, “dim” = dimension. **Right panel**: Depiction of calculation of Cramér's *V* between clinical group ∈{1,2} and individual-level feature clusters (groups) ∈{12,3}. *Top*: There is a perfect association between clinical group 1 and feature group 1, as well as a near-perfect association between clinical group 2 and feature group 2, and a perfect association between clinical group 2 and feature group 3. This yields a strong *V* (0.0.717). *Bottom*: The clinical group labels are scrambled, and this removes the shared information between clinical and feature label sets. Cramer's V goes to 0 as a result. Effect size interpretation of Cramér's *V* follows Kim (2017)^46^: for (rows-1) × (columns-1) degrees of freedom, i.e., (2–1) × (3-1) = 2 degrees of freedom, a value of *V* ≥ 0.35 is considered a large effect size. This approach would be applied to every combination of clinical variable and feature prediction (i.e., eight clinical variables × 53 acoustic features = 424 unique combinations). Grp. = group, Feat. = feature., a.u. = arbitrary units.

As a first analysis step after training BasisVAE, we determined whether embeddings captured clinical/demographic subgroups. We did this by statistically comparing subsets of the embeddings that corresponded to each clinical subgroup. Comparison was performed using the Fasano–Franceschini (FF) test, which is a multidimensional generalization of the Kolmogorov–Smirnov test.^44^ This analysis was implemented using the fasano.franceschini.test R (v 4.1.3) package.^45^ Bonferroni correction for 24 tests (eight measures × three tasks) yielded a significance threshold of *p* = 0.0021; eight measures included sex, site of onset, as well as the six clinical scores. See the left panel of Figure 1 for a visual depiction of this process.

As a second step, we extracted feature clusters from BasisVAE in order to determine which features were deemed to behave similarly when mapping latent data to predictions. The output consisted of probabilities of each feature belonging to each cluster. We set the value to extract at 3 initially, as we determined that 4 was redundant – setting the cluster number to 4 often resulted in an empty cluster, suggesting a low likelihood of this being a true model specification given the data. Notably, it was still possible to end up with less than three clusters if the likelihood of a subset of features being separate from others was low enough.

As a third step, we quantified the similarity of the model's predictions to clinical outcomes, in order to evaluate what the model learned at a more granular level. BasisVAE generated continuous-valued outputs that were tightly grouped, so we discretized them using Jenks’ method, with a number of groups determined using goodness-of-fit >90% (two to four groups were found in this way per output). Association between feature categories and clinical categories was determined using Cramér's *V*,^47^ depicted in the right panel of Figure 1. This yielded 424 values (eight clinical or demographic measures × 53 acoustic features).

Finally, for our fourth step, we clustered the matrix of Cramér's *V* values to explore patterns of association between clinical measures and acoustic features. This enabled us to determine whether embeddings captured meaningful clinical constructs, such as bulbar or respiratory function. This was done using the Python Seaborn package's *clustermap* function.

All machine learning and statistical analyses were performed using Python 3.8.9 and a combination of standardized packages (as specified above) and custom scripts.

## Results

### Analytical validity

For all tasks, we observed high correlations between corresponding features in both datasets. For example, in Bamboo, the measure of local absolute jitter existed in the output of both pipelines, and the rank correlation was strong (*ρ* = 0.899). For Phonation, the correlation for mean fundamental frequency (F0) in each case was strong (*ρ* = 0.892). Finally, for Winterlight's speech rate feature and the in-lab DDK-rate feature, the correlation was very strong (*ρ* = 0.927). In total, for Bamboo, out of 4240 unique correlations (80 in-lab features × 53 Winterlight features), 275 were “moderate,” 124 were “strong,” and 36 were “very strong.” For Phonation, these values were 98 (moderate), 20 (strong), and 0 (very strong), respectively, out of 2279 total correlations. Finally, for DDK, these values were 14 (moderate), 6 (strong), and 4 (very strong), respectively, out of 106 total correlations. Strong correlations across all tasks were typically observed for jitter, shimmer, timing, and F0 measures. Notably, most of the moderate (or lower) correlations were observed between features that did not directly correspond, e.g., F0 mean × speech rate.

**Figure 2.**
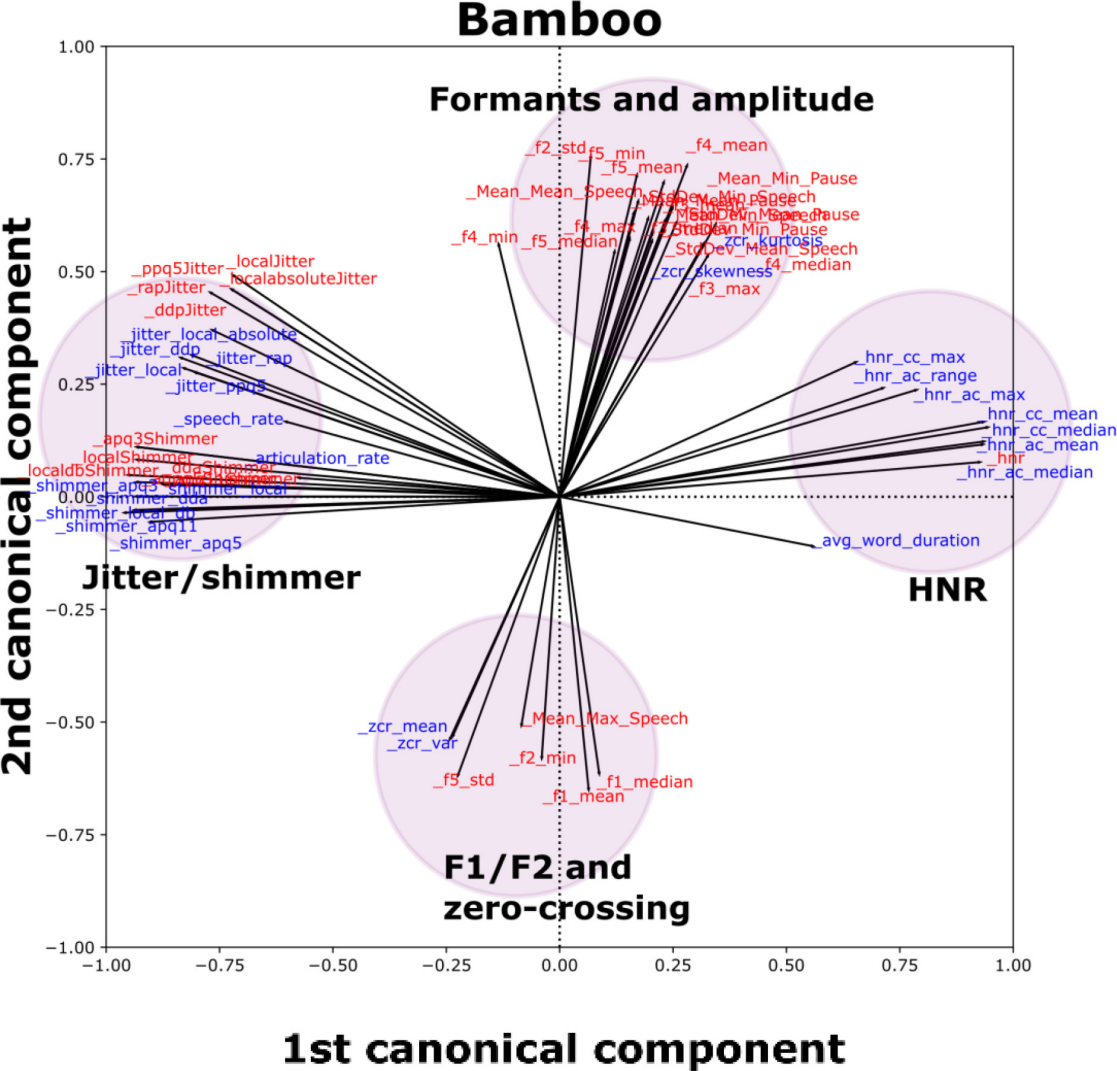
SCCA biplot for the Bamboo task (see Supplemental Figure S1 for other tasks), including features from in-lab and Winterlight feature sets with at least one loading (*x* or *y* axis) >|0.50|. Each individual vector (black line emanating from the origin) is associated with an individual feature. Groups of similar features are indicated by colored and shaded circles to make identification of clusters easier. Red features correspond to the in-lab dataset, and blue features correspond to the Winterlight feature set.

Figure 2 depicts the results of multivariate SCCA analyses. For each of the three tasks, there was a clear overlap between similar groups of features from the Winterlight and in-lab datasets. For Bamboo, we observed that salient features clustered together in the loading space; for example, jitter and shimmer features were grouped together on the left of the plot with similar orientations and magnitudes. Similar patterns were observed in Phonation (jitter/shimmer/HNR cluster, as well as an F0 and intensity cluster) and DDK (rate cluster and variability cluster) tasks, which are depicted in Supplemental Figure S1.

### Clinical validity

We observed relatively few moderate or strong univariate Spearman correlations between clinical measures and Winterlight features, but those that were observed were expected. For example, for Bamboo, we observed strong correlations between SIT-R and speech rate (*ρ* = 0.884), articulation rate (*ρ* = 0.881), and average word duration (*ρ* = -0.884). For DDK, we also observed strong correlations between FRS-bulb/SIT-R and measures of rate. For example, “speech rate” vs FRS-bulb was *ρ* = 0.640 and “articulation rate” vs SIT-R was *ρ* = 0.539, respectively. Finally, correlations in the Phonation task tended to center on relationships with sex and F0. For example, the sex vs F0 mean (*ρ* = 0.679) and %FVC vs F0 range (*ρ* = -0.426).

**Figure 3.**
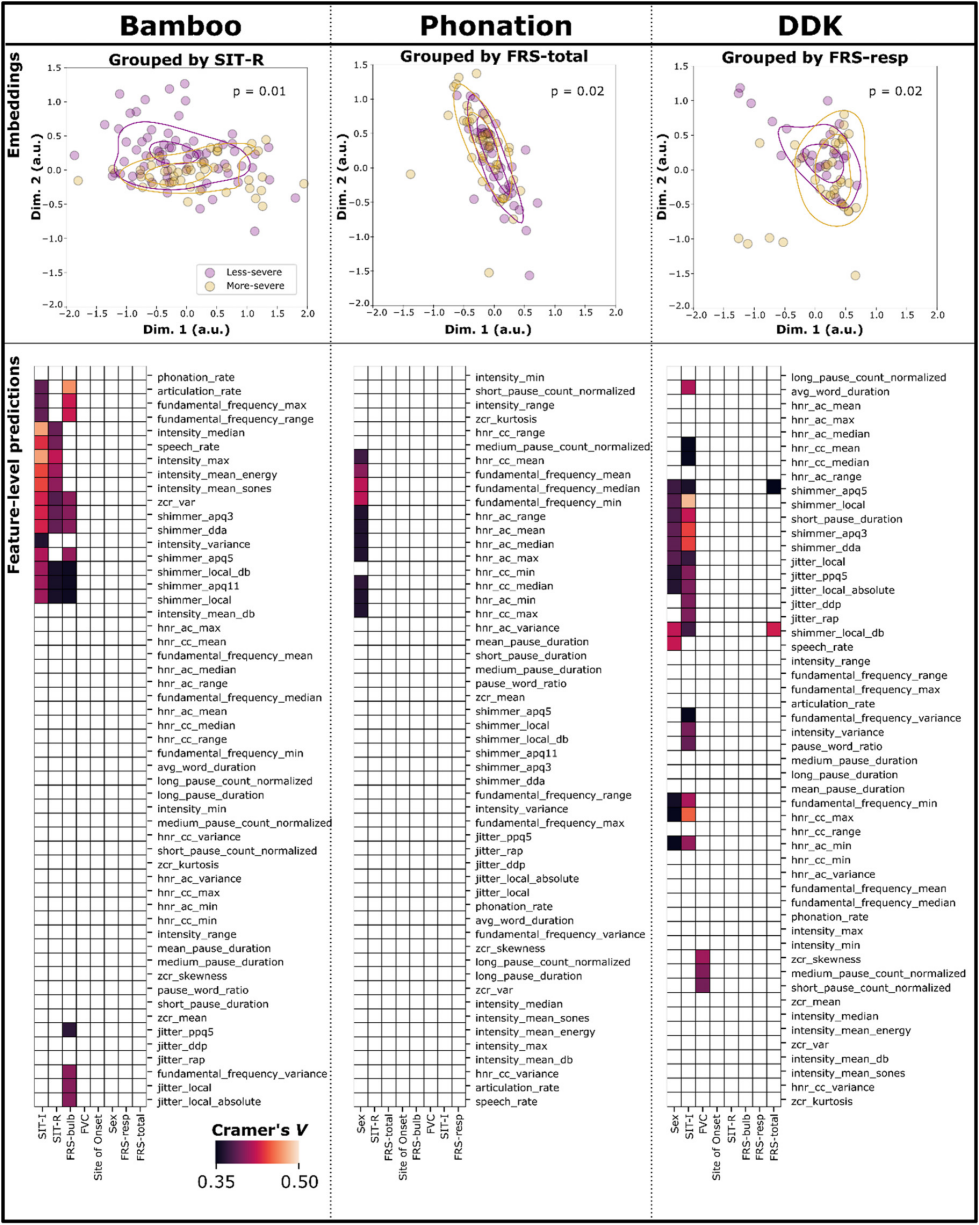
Summary of embedding results, feature-level predictions, and their correlations with clinical measures “bamboo,” “phonation,” and “DDK” applies to both plots in their respective columns. Top row (“embeddings”): Embeddings color-coded by individual clinical measures. Axes are identical across all three plots, and the legend in the Bamboo plot is valid for all three plots. Axes are in arbitrary units (a.u.). Bottom row (“feature-level predictions”): Heatmaps depicting clusters of Cramer's *V* values, with clustering performed across both clinical measures (horizontal axes) and acoustic feature values as predicted from BasisVAE (vertical axes). Each heatmap comprises all combinations of acoustic feature × clinical measures. Categorical correlations (Cramér's *V*) are thresholded between values of >0.35 (strong effect size) and <0.50 (to aid with visualization); i.e., cells that are white had categorical correlations ≤0.35. The color bar in the Bamboo heatmap plot applies to all three heatmaps.

BasisVAE embeddings captured notable differences between less-severe and more-severe clinical subgroups across all three tasks. The summary of statistical differences between these subgroups is summarized in Table 2 as FF test distance estimates. For example, the more-severe and less-severe subspaces of the Bamboo embeddings for SIT-I trended toward significant differences. Scatterplots in the top row of Figure 3 depict some example subgroupings for each of Bamboo, Phonation, and DDK tasks, color-coded by less-severe (purple) or more-severe (yellow) status. The clinical features depicted were selected based on the largest differences between subgroups on the FF test. We excluded the case of sex, as it is trivially strongly related to features in the Phonation task. For Phonation, we instead focused on FRS-total as a more classical “clinical measure” of interest. In all three cases, a trend toward a significant difference was observed (i.e., *p *< 0.05 but was not significant after Bonferroni correction). These subgroup differences were visually apparent for SIT-R/Bamboo (*p* = 0.01), with slightly lower subgroup differences observed in FRS-total/Phonation (*p* = 0.02) and FRS-resp/DDK (*p* = 0.02). Phonation and DDK embeddings also captured a trend toward a sex difference (*p* < 0.05), whereas Bamboo embeddings did not.

**Table 2.** FF test: distances between subgroup embedding distributions.

	Bamboo	Phonation	DDK
Site of onset	**1.50**	1.02	1.03
Sex	1.51	**1.54**	**1.82**
SIT-I	**2.14**	0.66	1.47
SIT-R	**2.35**	1.02	1.04
FRS-resp	1.67	1.28	**1.63**
FRS-bulb	**1.68**	0.68	0.83
FRS-total	1.44	**1.16**	1.23
%FVC	1.20	0.66	1.10

**Bold** indicates *p *< 0.05.

The automatic clustering of acoustic features generated by BasisVAE is summarized in Table 3. For Bamboo, for example, Cluster 1 consisted of mostly jitter, shimmer, rate-related features (articulation rate and speech rate), and pause counts. Cluster 2 consisted largely of HNR features as well as F0 mean/median and some pause duration measures. Finally, Cluster 3 consisted of mostly intensity features (as well as HNR max and range). For Phonation, Cluster 1 consisted primarily of jitter and shimmer. Cluster 2 consisted primarily of F0 (except max and range) and HNR measures. Finally, Cluster 3 consisted of only a few features such as rate-related ones (that are not meaningful for Phonation) as well as F0 max and range. Finally, for DDK, Cluster 1 consisted primarily of jitter and intensity measures, as well as min/max/range F0 measures. In contrast, Cluster 2 consisted mainly of F0 (mean/median/variance), HNR, and rate measures. Interestingly, for DDK, different rate measures associated with different clusters (Cluster 1, articulation rate; Cluster 2, “speech rate” and “average word duration,” which are indices of syllable repetition rate and average syllable length, respectively). Furthermore, BasisVAE analysis of DDK did not output a third cluster.

**Table 3.** Feature clusters derived from BasisVAE.

Bamboo	Phonation	DDK
articulation rate	avg word dur	articulation rate
F0 max	int min	F0 max
F0 range	int range	F0 min
F0 var	int var	F0 range
HNR cc var	jitter DDP	HNR ac min
int mean dB	jitter local	HNR cc max
int min	jitter local abs	int max
int var	jitter PPQ5	int mean dB
jitter DDP	jitter rap	int mean energy
jitter local	long pause ct norm	int mean sones
jitter local abs	long pause dur	int median
jitter PPQ5	med pause ct norm	int min
jitter rap	phonation rate	int range
med pause ct norm	shimmer APQ11	int var
shimmer APQ11	shimmer APQ3	jitter DDP
shimmer APQ3	shimmer APQ5	jitter local
shimmer APQ5	shimmer DDA	jitter local abs
shimmer DDA	shimmer local	jitter rap
shimmer local	shimmer local dB	med pause dur
shimmer local dB	short pause ct norm	pause word ratio
short pause ct norm	ZCR kurtosis	ZCR mean
speech rate	ZCR skewness	ZCR var
ZCR mean	F0 mean	avg word dur
ZCR var	F0 median	F0 mean
avg word dur	F0 min	F0 median
F0 mean	F0 var	F0 var
F0 median	HNR ac max	HNR ac max
F0 min	HNR ac mean	HNR ac mean
HNR ac max	HNR ac median	HNR ac median
HNR ac mean	HNR ac min	HNR ac range
HNR ac median	HNR ac range	HNR ac var
HNR ac min	HNR ac var	HNR cc mean
HNR ac range	HNR cc max	HNR cc median
HNR ac var	HNR cc median	HNR cc min
HNR cc mean	HNR cc min	HNR cc range
HNR cc median	HNR cc range	HNR cc var
HNR cc min	int max	jitter PPQ5
int range	int mean dB	long pause ct norm
long pause ct norm	int mean energy	long pause dur
long pause dur	int mean sones	mean pause dur
mean pause dur	int median	med pause ct norm
med pause dur	mean pause dur	phonation rate
pause word ratio	med pause dur	shimmer APQ3
phonation rate	pause word ratio	shimmer APQ5
short pause dur	short pause dur	shimmer DDA
ZCR kurtosis	articulation rate	shimmer local
ZCR skewness	F0 max	shimmer local dB
HNR cc max	F0 range	short pause ct norm
HNR cc range	HNR cc mean	short pause dur
int max	HNR cc var	speech rate
int mean energy	speech rate	ZCR kurtosis
int mean sones	ZCR mean	ZCR skewness
int median	ZCR var	shimmer APQ11

“ac” = autocorrelation, “cc” = cross-correlation, “ct” = count, “dur” = duration, “int” = intensity, “norm” = normalized, “max” = maximum, “min” = minimum. Colors are arbitrary and denote different clusters. Shimmer APQ11 (DDK; shown as a white cell) was removed from BasisVAE analysis because of missing values.

Feature-level predictions generated from BasisVAE often had strong and interpretable correlations with clinical measures, and clusters of these correlations further highlighted that the trained models learned meaningful mappings from acoustic features to embeddings. Heatmaps showing clusters of correlation values (distinct from the clustering generated by BasisVAE, above) are depicted in Figure 3 (bottom row). When the model was trained using Bamboo data, there was a clear group of feature-level predictions and clinical measures that correlated well (top left of the panel). Specifically, predictions of shimmer, intensity, and rate correlated strongly with clinical measures SIT-R, SIT-I, and FRS-bulb. When the model was trained using Phonation data, the only strong associations between acoustic feature predictions and clinical measures/demographics were found between F0/HNR predictions and sex. Finally, when the model was trained using DDK data, the strongest associations were found between predictions of timing (average word/syllable duration, pause/word/syllable ratio, etc.), jitter, and shimmer and SIT-I. Some of these predictions also had strong correlations with sex, except for the timing measures. Notably, short and medium pause count predictions were found to correlate strongly with %FVC.

## Discussion

In the present study, we validated Winterlight's automated speech analysis pipeline in a population of individuals with ALS. First, we determined the analytical validity of Winterlight's acoustic features by showing that they strongly corresponded to their lab-based counterparts. Next, we determined that Winterlight's acoustic features had moderate to strong relationships with relevant clinical measures. Altogether, we comprehensively demonstrated the analytical and clinical validity of Winterlight's acoustic feature set in ALS, in line with best practices as outlined in the V3 framework.^22^

Our analytical validity results highlighted that Winterlight's acoustic analysis pipeline captured similar aspects of speech to our lab-based pipeline, which is an important component of validation.^22^ The SCCA results strongly supported the univariate results. For example, jitter features between Winterlight and in-lab analyses typically correlated well with each other, and additionally they grouped together in the multivariate analyses. These patterns of association between groups of features typically held across multiple tasks, although the extent of this varied slightly across tasks. For example, the groups of jitter and shimmer features for Winterlight and in-lab analyses overlapped in the Bamboo task, whereas they were separated in the Phonation task, albeit not very far. Potentially, this was related to differences in preprocessing or specific sections of audio that were selected for analysis. However, given that these features have previously shown utility in ALS,^7–12^ this provided a strong support for subsequent clinical validation.

In addition to promising analytical validation results, we also demonstrated the clinical validity of Winterlight's pipeline by distinguishing more-severe and less-severe subgroups of ALS and capturing meaningful univariate relationships between clinical measures and acoustic features. For example, we observed moderate or strong correlations between SIT-R and speaking rate (Bamboo), between FRS-bulb and PPQ5-jitter (Bamboo), and between DDK-rate and “speech rate” (DDK). These are either expected (rate-based measures) or supported by previous literature (e.g., SIT-R and automated estimate of speaking rate^28^). The lack of strong correlations in Phonation for any features other than sex × F0 features was surprising, given that the previous work has identified jitter, shimmer, and other acoustic features as valuable indicators of ALS disease presence vs healthy controls.^48^ It is possible that our severity thresholds were too conservative to detect a difference between subgroups using individual features or that the effects of sex differences overwhelmed remaining signal. Furthermore, it is possible that some of our patients were less-affected in their phonatory systems than their articulatory subsystems at the time of assessment. Future work could explore subgroup analyses of male and female participants to investigate this possibility further. Nevertheless, we identified strong correlations between meaningful features in the Bamboo and DDK tasks, suggesting that these task types may be more robust to this type of confounding. Future work should seek to perform detailed longitudinal analyses of changes in individual speech features that correspond to, e.g., clinical constructs of speaking rate and intelligibility, although this is out of scope for the present study.

Our multivariate clinical validation analyses largely supported and expanded upon the univariate analyses. For example, when BasisVAE was trained using the Phonation data, it was able to detect a trend toward significance between FRS-total severity subgroups and embeddings (*p* = 0.02). Given that the FRS-total score is multidimensional,^49^ this could indicate that individual acoustic features would not inherently be able to capture variability in this scale. Further to this, no individual feature-level predictions derived from embeddings had strong correlations with FRS-total, nor any clinical/demographic measure aside from sex, which agreed with the results of our univariate Spearman correlations. Taken together, this suggests potentially that Phonation data was not easily characterized by only a few key features in the present dataset. Notably, in an effort to avoid biasing our approach, we retained all features from Winterlight's pipeline or multivariate analyses. Future work should likely focus on specific feature subsets for characterizing simpler tasks such as Phonation or DDK. This contrasts with observations for Bamboo data, where feature-level predictions of rate-related features (speech rate, articulation rate, etc.) were strongly correlated with FRS-bulb as well as SIT-I and SIT-R. Additional relationships were found between these clinical measures of bulbar disease severity and feature-level predictions for jitter and shimmer. These have been found to relate to ALS severity previously.^50,51^ These results point the way toward possible use cases for identifying relationships between multivariate acoustic feature groups and multidimensional clinical scales. For example, composite scores might capture larger amounts of information across multiple speech domains in an interpretable way. A composite feature approach could potentially address the findings of relatively few univariate strong correlations that were observed in this study.

Given that acoustic assessment has proven valuable for detecting early bulbar symptoms in patients with ALS,^52^ there has been substantial interest in developing speech assessment tools for ALS clinical trials and research purposes. Other beneficial properties of speech assessments include the brevity of assessments and potential for scalable remote deployment. Furthermore, novel biomarkers, such as digital/speech biomarkers, are potentially valuable ways to overcome limitations imposed by clinical heterogeneity that have so far confounded ALS clinical trials.^53^ However, up to this point, the adoption of digital biomarkers has been limited by a lack of validation and inconsistent evaluation practices,^54–56^ despite the increased use of connected digital health products in clinical research.^57^ A structured validation approach such as V3 is important for the eventual incorporation of digital speech assessment technologies into clinical research and clinical trials,^58^ which inspired the structure of the present study. Future validation work in the realm of ALS will include further exploration of the utility of Winterlight's analysis system for not only motor speech/acoustic but also cognitive features, which will address an important unmet need given the prevalence of cognitive/linguistic impairments in ALS.^59^ It additionally may be of value to consider the comparison of multiple speech and language assessment tools, in order to identify their strengths and limitations in assessing motor speech and cognitive deficits in ALS and other neurodegenerative diseases. This could also involve validation of Winterlight's cognitive features specifically in ALS patients, given the spectrum of cognitive impairments that occur along the ALS-FTD continuum.

Our study has some limitations to consider. First, in the interest of brevity, we did not explore interactions of acoustic features across multiple tasks. This may provide interesting, high-level perspective on the behavior of acoustic features. Because our current multivariate methods (sparse CCA and a VAE) both scale well to settings with more features than samples, these objectives can be readily handled in subsequent work, where the full scope can be dedicated to cross-task analyses. Additionally, Winterlight's feature set consists primarily of non-acoustic features, such as linguistic/cognitive indicators that have been explored in great depth in previous studies. Although the present tasks (Bamboo, Phonation, and DDK) were not designed to exploit these features, future ALS research using their pipeline may consist of open-ended speaking tasks or fluency tasks. These cognitive–intensive tasks may also serve to further highlight the dichotomization between bulbar and spinal onset cases, given the overlap between bulbar-onset ALS and cognitive impairment.^60^ Regarding our choice of preprocessing, it will be important in future work to determine any adverse effects of noise suppression on spectral and cepstral features. Many of the features that were most robustly associated with clinical features in the present study, e.g., rate-related/pause-related features, would be expected to be unaffected by this type of processing. Others such as jitter and shimmer may be more so. We leave this analysis to future work, but note that the features that were found related to clinical outcomes were sensible and supported by previous literature. Furthermore, future work should explore wider ranges of scores in clinical features. The present sample was biased toward less-severe presentations, e.g., the median FRS-bulb score was 11/12, which indicates a small deviation from healthy levels of functioning. Our sample also had some missing clinical data; although the majority of participants had clinical measures (≥100 participants in most cases), there was some variation in the amount across different measures. Future prospective studies will be better-equipped to address this shortcoming. Related to this, a formal power calculation was not performed for the present study. We reasoned that our sample size was adequate given the exploratory nature of this work, but future studies should incorporate such analyses. Additionally, for our clinical Winterlight feature univariate correlations, we did not adjust for multiple comparisons and instead chose a magnitude-based interpretation. This is related to many of the correlations not having meaningful associations (e.g., measures of pausing and rate have no meaningful relationship with the Phonation task), and so we did not wish to dilute true effects using an overly punishing correction factor that includes correlations we would not expect to be strong anyway. Furthermore, future work should explore similar analyses on remote recordings to provide additional support for the current findings and to explore the possible contributions of different types of recording equipment.

## Conclusions

In this study, we sought to determine the analytical and clinical validity of Winterlight's acoustic feature set in a large population of individuals with ALS. We determined (1) that this acoustic feature set demonstrated analytical validity with respect to a lab-based feature set and (2) that it was able to capture relevant clinical phenomena. Future work will seek to build off of these promising results, in an effort to develop robust and sensitive speech analysis tools for use as digital endpoints in a variety of research contexts and clinical trials.

## Supplemental Material

sj-docx-1-dhj-10.1177_20552076231219102 - Supplemental material for Validation of automated pipeline for the assessment of a motor speech disorder in amyotrophic lateral sclerosis (ALS) Click here for additional data file. Supplemental material, sj-docx-1-dhj-10.1177_20552076231219102 for Validation of automated pipeline for the assessment of a motor speech disorder in amyotrophic lateral sclerosis (ALS) by Leif ER Simmatis, Jessica Robin, Timothy Pommée, Scotia McKinlay, Rupinder Sran, Niyousha Taati, Justin Truong, Bharatkumar Koyani and Yana Yunusova in DIGITAL HEALTH
