# Supplementary material for: Validation of automated pipeline for the assessment of a motor speech disorder in amyotrophic lateral sclerosis (ALS)
Source: Digit Health. 2023 Dec 21;9:20552076231219102. doi: 10.1177/20552076231219102 (PMC10748679; doi:10.1177/20552076231219102)
Supplement: sj-docx-1-dhj-10.1177_20552076231219102 - Supplemental material for Validation of automated pipeline for the assessment of a motor speech disorder in amyotrophic lateral sclerosis (ALS) [file sj-docx-1-dhj-10.1177_20552076231219102.docx]

**Supplemental**


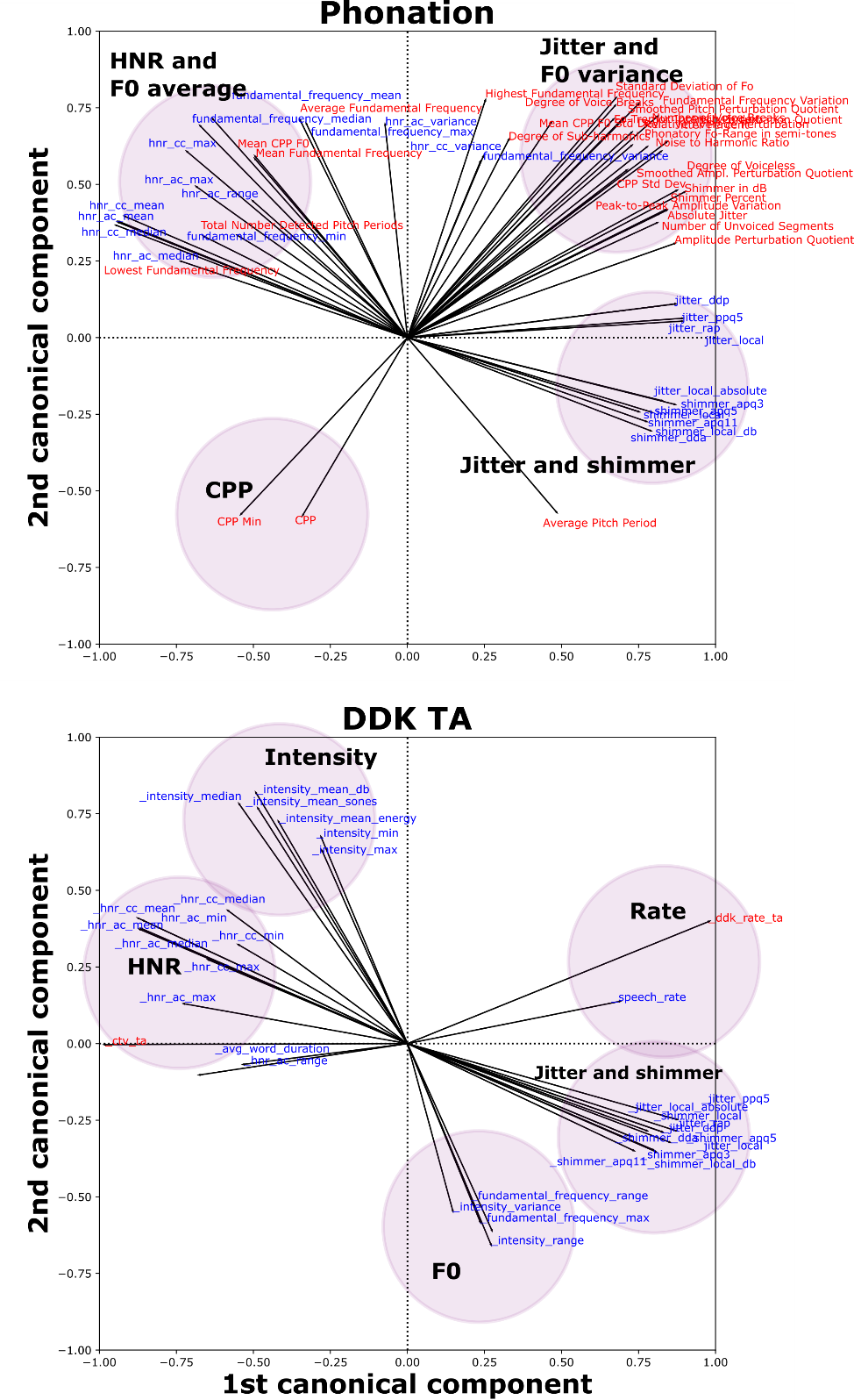


Figure S1. SCCA biplots for Phonation and DDK tasks (cf. Figure 2 in the main article), including features from in-lab and Winterlight feature sets with at least one loading (x or y axis) >|0.50|. Each individual vector (black line emanating from the origin) is associated with an individual feature. Groups of similar features are indicated by coloured and shaded circles to make identification of clusters easier.

**Supplemental Table 1.** Acoustic features and their descriptions (Winterlight pipeline).

| Feature name | Description |
| --- | --- |
| fundamental_frequency_max | The max of the sequence of fundamental frequency values extracted from the audio file, using the Parselmouth library (equivalent to Praat method for computing fundamental frequency). In Hz. The cutoff range is 70-620 Hz. |
| fundamental_frequency_mean | The mean of the sequence of fundamental frequency values extracted from the audio file, using the Parselmouth library (equivalent to Praat method for computing fundamental frequency). In Hz. The cutoff range is 70-620 Hz. |
| fundamental_frequency_median | The median of the sequence of fundamental frequency values extracted from the audio file, using the Parselmouth library (equivalent to Praat method for computing fundamental frequency). In Hz. The cutoff range is 70-620 Hz. |
| fundamental_frequency_min | The min of the sequence of fundamental frequency values extracted from the audio file, using the Parselmouth library (equivalent to Praat method for computing fundamental frequency). In Hz. The cutoff range is 70-620 Hz. |
| fundamental_frequency_range | The difference between the maximum and minimum fundamental frequency value extracted from the audio file. Computed as (fundamental_frequency_max - fundamental_frequency_min). In Hz. The cutoff range is 70-620 Hz. |
| fundamental_frequency_variance | The variance of the sequence of fundamental frequency values extracted from the audio file, using Praat. In Hz. The cutoff range is 70-620 Hz. |
| hnr_ac_max | The max degree of acoustic periodicity, in dB, using the autocorrelation method. The ratio of energy between the signal in the periodic part and the noise (e.g. An HNR of 0 dB means there is equal energy in the harmonics and the noise). |
| hnr_ac_mean | The mean degree of acoustic periodicity, in dB, using the autocorrelation method. The ratio of energy between the signal in the periodic part and the noise (e.g. An HNR of 0 dB means there is equal energy in the harmonics and the noise). |
| hnr_ac_median | The median degree of acoustic periodicity, in dB, using the autocorrelation method. The ratio of energy between the signal in the periodic part and the noise (e.g. An HNR of 0 dB means there is equal energy in the harmonics and the noise). |
| hnr_ac_min | The min degree of acoustic periodicity, in dB, using the autocorrelation method. The ratio of energy beteween the signal in the periodic part and the noise (e.g. An HNR of 0 dB means there is equal energy in the harmonics and the noise). |
| hnr_ac_range | The range in degree of acoustic periodicity, in dB, using the autocorrelation method. The ratio of energy beteween the signal in the periodic part and the noise (e.g. An HNR of 0 dB means there is equal energy in the harmonics and the noise). |
| hnr_ac_variance | The variance in the degree of acoustic periodicity, in dB, using the autocorrelation method. The ratio of energy beteween the signal in the periodic part and the noise (e.g. An HNR of 0 dB means there is equal energy in the harmonics and the noise). |
| hnr_cc_max | The max degree of acoustic periodicity, in dB, using the cross-correlation method. The ratio of energy beteween the signal in the periodic part and the noise (e.g. An HNR of 0 dB means there is equal energy in the harmonics and the noise). |
| hnr_cc_mean | The mean degree of acoustic periodicity, in dB, using the cross-correlation method. The ratio of energy beteween the signal in the periodic part and the noise (e.g. An HNR of 0 dB means there is equal energy in the harmonics and the noise). |
| hnr_cc_median | The median degree of acoustic periodicity, in dB, using the cross-correlation method. The ratio of energy beteween the signal in the periodic part and the noise (e.g. An HNR of 0 dB means there is equal energy in the harmonics and the noise). |
| hnr_cc_min | The min degree of acoustic periodicity, in dB, using the cross-correlation method. The ratio of energy beteween the signal in the periodic part and the noise (e.g. An HNR of 0 dB means there is equal energy in the harmonics and the noise). |
| hnr_cc_range | The range in degree of acoustic periodicity, in dB, using the cross-correlation method. The ratio of energy beteween the signal in the periodic part and the noise (e.g. An HNR of 0 dB means there is equal energy in the harmonics and the noise). |
| hnr_cc_variance | The variance in the degree of acoustic periodicity, in dB, using the cross-correlation method. The ratio of energy beteween the signal in the periodic part and the noise (e.g. An HNR of 0 dB means there is equal energy in the harmonics and the noise). |
| intensity_max | The maximum of the intensity curve (i.e. perceived loudness), relative to 2*10^-5 Pa (normative auditory threshold for a 1000-Hz sine wave). In dB. |
| intensity_mean_db | The mean of the intensity curve (i.e. perceived loudness), relative to 2*10^-5 Pa (normative auditory threshold for a 1000-Hz sine wave). In dB. |
| intensity_mean_energy | The mean of the intensity curve (i.e. perceived loudness), relative to 2*10^-5 Pa (normative auditory threshold for a 1000-Hz sine wave). In Pa2/s (i.e. the mean power) |
| intensity_mean_sones | The mean of the intensity curve (i.e. perceived loudness), relative to 2*10^-5 Pa (normative auditory threshold for a 1000-Hz sine wave), based on the averaging properties of the human ear. In sones. |
| intensity_median | The median of the intensity curve (i.e. perceived loudness), relative to 2*10^-5 Pa (normative auditory threshold for a 1000-Hz sine wave). In dB. |
| intensity_min | The minimum of the intensity curve (i.e. perceived loudness), relative to 2*10^-5 Pa (normative auditory threshold for a 1000-Hz sine wave). In dB. |
| intensity_range | The difference between the maximum and minimum of the intensity curve (i.e. percieved loudness), relative to 2*10^-5 Pa (normative auditory threshold for a 1000-Hz sine wave). In dB. |
| intensity_variance | The variance of the intensity curve (i.e. perceived loudness), relative to 2*10^-5 Pa (normative auditory threshold for a 1000-Hz sine wave). In dB. |
| jitter_ddp | Average absolute difference between consecutive differences between consecutive periods, divided by the average period. The value is three times RAP. A percentage. For the precise procedure, see: http://www.fon.hum.uva.nl/praat/manual/Voice_2__Jitter.html |
| jitter_local | The average absolute difference between consecutive periods, divided by the average period. A percentage. For the precise procedure, see: http://www.fon.hum.uva.nl/praat/manual/Voice_2__Jitter.html |
| jitter_local_absolute | The average absolute difference between consecutive periods, in seconds. For the precise procedure, see: http://www.fon.hum.uva.nl/praat/manual/Voice_2__Jitter.html |
| jitter_ppq5 | Five-point Period Perturbation Quotient, the average absolute difference between a period and the average of it and its four closest neighbours, divided by the average period. A percentage. For the precise procedure, see: http://www.fon.hum.uva.nl/praat/manual/Voice_2__Jitter.html |
| jitter_rap | Relative Average Perturbation, the average absolute difference between a period and the average of it and its two neighbours, divided by the average period. A percentage. For the precise procedure, see: http://www.fon.hum.uva.nl/praat/manual/Voice_2__Jitter.html |
| long_pause_count_normalized | Number of pauses > 2 sec, divided by the length of audio in sec |
| long_pause_duration | The total duration of pauses (unvoiced segments) > 2 sec, in seconds |
| mean_pause_duration | The duration of unvoiced segments divided by total number unvoiced segments (VAD). Includes *ALL* unvoiced segments (including < 150 ms). |
| medium_pause_count_normalized | Number of pauses 1-2 sec, divided by the length of audio in sec |
| medium_pause_duration | The total duration of pauses (unvoiced segments) that are 1-2 sec, in seconds |
| pause_word_ratio | Number of unvoiced segments longer than 150 ms divided by number of voiced segments (comes from VAD module). |
| phonation_rate | Number of voiced samples (50ms windows) over the total number of samples |
| shimmer_apq11 | The 11-point Amplitude Perturbation Quotient, the average absolute difference between the amplitude of a period and the average of the amplitudes of it and its ten closest neighbours, divided by the average amplitude. A percentage. |
| shimmer_apq3 | The three-point Amplitude Perturbation Quotient, the average absolute difference between the amplitude of a period and the average of the amplitudes of its neighbours, divided by the average amplitude. A percentage. |
| shimmer_apq5 | The five-point Amplitude Perturbation Quotient, the average absolute difference between the amplitude of a period and the average of the amplitudes of it and its four closest neighbours, divided by the average amplitude. A percentage. |
| shimmer_dda | The average absolute difference between consecutive differences between the amplitudes of consecutive periods. A percentage. |
| shimmer_local | The average absolute difference between the amplitudes of consecutive periods, divided by the average amplitude. A percentage. |
| shimmer_local_db | The average absolute base-10 logarithm of the difference between the amplitudes of consecutive periods. In dB. |
| short_pause_count_normalized | Number of pauses < 1 sec (no bottom limit! review!), divided by the length of audio in sec |
| short_pause_duration | The total duration of pauses (unvoiced segments) < 1 sec (no bottom limit! review!), in seconds |
| zcr_kurtosis | Kurtosis of the number of zero crossings (changes from positive to negative or negative to positive signal value) across all voiced frames |
| zcr_mean | Mean number of zero crossings (changes from positive to negative or negative to positive signal value) across all voiced frames |
| zcr_skewness | Skewness of the number of zero crossings (changes from positive to negative or negative to positive signal value) across all voiced frames |
| zcr_var | Variance of the number of zero crossings (changes from positive to negative or negative to positive signal value) across all voiced frames |
| articulation_rate | The total number of syllables for words in the transcript (note: "xxx" unintelligible words are counted as having 1 syllable, based on the average number of word syllables in an internal dataset) divided by the total duration of voiced audio (i.e. speech). In number of syllables per second. The value will be 0 if there are no words/speech in the audio. |
| avg_word_duration | The length of audio (in seconds) divided by the number of words in transcript |
| speech_rate | Number of words per minute |

**Supplemental Table 2.** Acoustic features and their descriptions (in-lab pipeline).

| Task | Feature name | Description |
| --- | --- | --- |
| Bamboo | PerPause | Total percent pause time in the signal. |
| Bamboo | PerSpeech | Total percent speech time in the signal. |
| Bamboo | Pause_Events | Total number of pause events in the signal. |
| Bamboo | Speech_Events | Total number of speech events in the signal. |
| Bamboo | Peak_Freq | Peak frequency. |
| Bamboo | Peak_Amp | Peak amplitude. |
| Bamboo | Three_dB | 3 decibel cutoff. |
| Bamboo | Mean_Pause | Mean of the DURATIONS of individual pause segments in msec. |
| Bamboo | Mean_Speech | Mean of the DURATIONS of individual speech segments in msec. |
| Bamboo | StdDev_Pause | Standard Deviation of the DURATIONS of individual speech segments in msec. |
| Bamboo | StdDev_Speech | Standard Deviation of the DURATIONS of individual pause segments in msec. |
| Bamboo | CV_Speech_Duration | Coefficient of variation - Ratio between stddev of speech duration and mean of speech duration. |
| Bamboo | CV_Pause_Duration | Coefficient of variation - Ratio between stddev of pause duration and mean of pause duration. |
| Bamboo | CVR | Coefficient of variation ratio - Ratio of CV_Speech and CV_Pause. |
| Bamboo | StdDev_AllSignal | Standard deviation of the AMPLITUDE of entire signal. |
| Bamboo | Mean_Min_Speech | Average of minimum AMPLITUDE of individual speech segments. |
| Bamboo | Mean_Max_Speech | Average of maximum AMPLITUDE of individual speech segments. |
| Bamboo | Mean_Mean_Speech | Average of mean AMPLITUDE of individual speech segments. |
| Bamboo | Mean_StdDev_Speech | Average of standard deviation of AMPLITUDE of individual speech segments. |
| Bamboo | StdDev_Min_Speech | Standard Deviation of minimum AMPLITUDE of individual speech segments. |
| Bamboo | StdDev_Max_Speech | Standard Deviation of maximum AMPLITUDE of individual speech segments. |
| Bamboo | StdDev_Mean_Speech | Standard Deviation of mean AMPLITUDE of individual speech segments. |
| Bamboo | StdDev_StdDev_Speech | Standard Deviation of standard deviation AMPLITUDE of individual speech segments. |
| Bamboo | CV_Min_Speech | Coeffieicnt of variation - Ratio between stddev_minimum_speech and mean_minimum_speech. |
| Bamboo | CV_Max_Speech | Coeffieicnt of variation - Ratio between stddev_maximum_speech and mean_maximum_speech. |
| Bamboo | CV_Mean_Speech | Coeffieicnt of variation - Ratio between stddev_mean_speech and mean_mean_speech. |
| Bamboo | CV_StdDev_Speech | Coeffieicnt of variation - Ratio between stddev_stddev_speech and mean_stddev_speech. |
| Bamboo | Mean_Min_Pause | Average of minimum AMPLITUDE of individual pause segments |
| Bamboo | Mean_Max_Pause | Average of maximum AMPLITUDE of individual pause segments |
| Bamboo | Mean_Mean_Pause | Average of mean AMPLITUDE of individual pause segments. |
| Bamboo | Mean_StdDev_Pause | Average of standard deviation of AMPLITUDE of individual pause segments. |
| Bamboo | StdDev_Min_Pause | Standard Deviation of minimum AMPLITUDE of individual pause segments. |
| Bamboo | StdDev_Max_Pause | Standard Deviation of maximum AMPLITUDE of individual pause segments. |
| Bamboo | StdDev_Mean_Pause | Standard Deviation of mean AMPLITUDE of individual pause segments. |
| Bamboo | Stddev_StdDev_Pause | Standard Deviation of standard deviation of AMPLITUDE of individual pause segments. |
| Bamboo | CV_Min_Pause | Coeffieicnt of variation - Ratio between stddev_minimum_pause and mean_minimum_pause. |
| Bamboo | CV_Max_Pause | Coeffieicnt of variation - Ratio between stddev_maximum_pause and mean_maximum_pause. |
| Bamboo | CV_Mean_Pause | Coeffieicnt of variation - Ratio between stddev_mean_pause and mean_mean_pause. |
| Bamboo | CV_StdDev_Pause | Coeffieicnt of variation - Ratio between stddev_stddev_pause and mean_stddev_speech. |
| Bamboo | rapJitter | Relative average perturbation jitter. |
| Bamboo | localJitter | Local jitter. |
| Bamboo | localabsoluteJitter | Local absolute jitter. |
| Bamboo | ppq5Jitter | 5-point pitch perturbation quotient jitter. |
| Bamboo | ddpJitter | Difference of difference of periods jitter. |
| Bamboo | localShimmer | Local shimmer. |
| Bamboo | localdbShimmer | Local decibel shimmer. |
| Bamboo | apq3Shimmer | 3-point amplitude perturbation quotient shimmer. |
| Bamboo | aqpq5Shimmer | 5-point amplitude perturbation quotient shimmer. |
| Bamboo | apq11Shimmer | 11-point amplitude perturbation quotient shimmer. |
| Bamboo | ddaShimmer | Difference of differences of amplitudes shimmer. |
| Bamboo | hnr | Harmonic to noise ratio. |
| Bamboo | mean_pitch | Mean fundamental frequency |
| Bamboo | sd_pitch | Standard deviation of fundamental frequency. |
| Bamboo | pitch_slope | Slope of fundamental frequency trajectory. |
| Bamboo | f1_mean | Mean of first formant. |
| Bamboo | f1_std | Standard deviation of first formant. |
| Bamboo | f1_median | Median of first formant. |
| Bamboo | f1_min | Minimum of first formant. |
| Bamboo | f1_max | Maximum of first formant. |
| Bamboo | f2_mean | Mean of second formant. |
| Bamboo | f2_std | Standard deviation of second formant. |
| Bamboo | f2_median | Median of second formant. |
| Bamboo | f2_min | Minimum of second formant. |
| Bamboo | f2_max | Maximum of second formant. |
| Bamboo | f3_mean | Mean of third formant. |
| Bamboo | f3_std | Standard deviation of third formant. |
| Bamboo | f3_median | Median of third formant. |
| Bamboo | f3_min | Minimum of third formant. |
| Bamboo | f3_max | Maximum of third formant. |
| Bamboo | f4_mean | Mean of fourth formant. |
| Bamboo | f4_std | Standard deviation of fourth formant. |
| Bamboo | f4_median | Median of fourth formant. |
| Bamboo | f4_min | Minimum of fourth formant. |
| Bamboo | f4_max | Maximum of fourth formant. |
| Bamboo | f5_mean | Mean of fifth formant. |
| Bamboo | f5_std | Standard deviation of fifth formant. |
| Bamboo | f5_median | Median of fifth formant. |
| Bamboo | f5_min | Minimum of fifth formant. |
| Bamboo | f5_max | Maximum of fifth formant. |
| Bamboo | artic_ent | Articulatory entropy. |
| Phonation | Average Fundamental Frequency | Average fundamental frequency. |
| Phonation | Mean Fundamental Frequency | Mean fundamental frequency. |
| Phonation | Average Pitch Period | Average pitch period. |
| Phonation | Highest Fundamental Frequency | Maximum fundamental frequency. |
| Phonation | Lowest Fundamental Frequency | Minimum fundamental frequency. |
| Phonation | Standard Deviation of Fo | Standard deviation of fundamental frequency. |
| Phonation | Phonatory Fo-Range in semi-tones | Phonatory fundamental frequency range in semitones. |
| Phonation | Fo-Tremor Frequency | Fundamental frequency tremor frequency. |
| Phonation | Amplitude Tremor Frequency | Amplitude of tremor frequency. |
| Phonation | Absolute Jitter | Absolute jitter. |
| Phonation | Jitter Percent | Jitter percent. |
| Phonation | Relative Average Perturbation | Relative average perturbation jitter. |
| Phonation | Pitch Perturbation Quotient | Pitch perturbation quotient jitter. |
| Phonation | Smoothed Pitch Perturbation Quotient | Smoothed pitch perturbation quotient jitter. |
| Phonation | Fundamental Frequency Variation | Variance of fundamental frequency. |
| Phonation | Shimmer in dB | Shimmer in decibels. |
| Phonation | Shimmer Percent | Shimmer in percent. |
| Phonation | Amplitude Perturbation Quotient | 11-point amplitude perturbation quotient shimmer. |
| Phonation | Smoothed Ampl. Perturbation Quotient | Smoothed amplitude perturbation quotient shimmer. |
| Phonation | Peak-to-Peak Amplitude Variation | Local shimmer. |
| Phonation | Noise to Harmonic Ratio | Noise to harmonic ratio. |
| Phonation | Voice Turbulence Index | Average ratio of spectral inharmonic high-frequency energy (2800-5800Hz) to spectral harmonic energy (70-4500Hz). |
| Phonation | Soft Phonation Index | Average ratio of low-frequency harmonic energy (70-1600Hz) to the higher-frequency energy (1600-4500Hz). |
| Phonation | Fo-Tremor Intensity Index | Average ratio of frequency magnitude of the most intense low-frequency modulating component to the total frequency magnitude. |
| Phonation | Amplitude Tremor Intensity Index | Average ratio of the amplitude of the strongest low-frequency amplitude-modulated component relative to total amplitude. |
| Phonation | Degree of Voice Breaks | Relative number of segments representing voice breaks. |
| Phonation | Degree of Sub-harmonics | Relative subharmonic components of voice signal. |
| Phonation | Degree of Voiceless | Relative amount of nonharmonic areas in the voice sample. |
| Phonation | Number of Voice Breaks | Number of times the fundamental period was interrupted during the voice sample. |
| Phonation | Number of Sub-harmonic Segments | Number of segments where pitch was found to be a subharmonic of the fundamental frequency. |
| Phonation | Number of Unvoiced Segments | Number of segments containing interrupted voicing during the voice sample. |
| Phonation | Number of Segments Computed | Total number of segments. |
| Phonation | Total Number Detected Pitch Periods |  |
| Phonation | CPP | Cepstral peak prominence. |
| Phonation | CPP Std Dev | Standard deviation of CPP. |
| Phonation | CPP Max | Maximum of CPP. |
| Phonation | CPP Min | Minimum of CPP. |
| Phonation | L/H Spectral Ratio | Low-to-high frequency spectral ratio. |
| Phonation | L/H Spectral Ratio Std Dev | Standard deviation of low-to-high frequency spectral ratio. |
| Phonation | L/H Spectral Ratio Max | Maximum of low-to-high frequency spectral ratio. |
| Phonation | L/H Spectral Ratio Min | Minimum of low-to-high frequency spectral ratio. |
| Phonation | Mean CPP F0 | Mean CPP of fundamental frequency. |
| Phonation | Mean CPP F0 Std Dev | Standard deviation of mean CPP of fundamental frequency. |
| DDK-TA | DDK rate | Mean rate (syllables/second) during DDK. |
| DDK-TA | CTV | Cycle-to-cycle variability of DDK syllable timing. |

**Supplemental Table 2.** Spearman rho values for strong (|rho|>0.70) and very strong (|rho|>0.90) feature pairs between in-lab and Winterlight feature sets, for each of the 3 tasks.

| **Task** | **Lab features** | **Winterlight features** | **Rho** |
| --- | --- | --- | --- |
| Bamboo | rapJitter | jitter_local | 0.71751 |
| Bamboo | rapJitter | jitter_local_absolute | 0.808954 |
| Bamboo | rapJitter | jitter_ppq5 | 0.735497 |
| Bamboo | localJitter | jitter_ddp | 0.712969 |
| Bamboo | localJitter | jitter_local | 0.786295 |
| Bamboo | localJitter | jitter_local_absolute | 0.861245 |
| Bamboo | localJitter | jitter_ppq5 | 0.792183 |
| Bamboo | localJitter | jitter_rap | 0.712969 |
| Bamboo | localabsoluteJitter | fundamental_frequency_mean | -0.77227 |
| Bamboo | localabsoluteJitter | fundamental_frequency_median | -0.78023 |
| Bamboo | localabsoluteJitter | jitter_local_absolute | 0.898712 |
| Bamboo | ppq5Jitter | jitter_local | 0.715043 |
| Bamboo | ppq5Jitter | jitter_local_absolute | 0.821078 |
| Bamboo | ppq5Jitter | jitter_ppq5 | 0.732542 |
| Bamboo | ddpJitter | jitter_local | 0.71751 |
| Bamboo | ddpJitter | jitter_local_absolute | 0.808954 |
| Bamboo | ddpJitter | jitter_ppq5 | 0.735497 |
| Bamboo | localShimmer | hnr_ac_mean | -0.82492 |
| Bamboo | localShimmer | hnr_ac_median | -0.82748 |
| Bamboo | localShimmer | hnr_cc_mean | -0.84206 |
| Bamboo | localShimmer | hnr_cc_median | -0.84616 |
| Bamboo | localShimmer | jitter_ddp | 0.775078 |
| Bamboo | localShimmer | jitter_local | 0.77289 |
| Bamboo | localShimmer | jitter_local_absolute | 0.743191 |
| Bamboo | localShimmer | jitter_ppq5 | 0.793873 |
| Bamboo | localShimmer | jitter_rap | 0.775078 |
| Bamboo | localShimmer | shimmer_apq11 | 0.92713 |
| Bamboo | localShimmer | shimmer_apq3 | 0.958211 |
| Bamboo | localShimmer | shimmer_apq5 | 0.965934 |
| Bamboo | localShimmer | shimmer_dda | 0.958211 |
| Bamboo | localShimmer | shimmer_local | 0.970039 |
| Bamboo | localShimmer | shimmer_local_db | 0.949461 |
| Bamboo | localdbShimmer | hnr_ac_mean | -0.83789 |
| Bamboo | localdbShimmer | hnr_ac_median | -0.83786 |
| Bamboo | localdbShimmer | hnr_cc_mean | -0.85315 |
| Bamboo | localdbShimmer | hnr_cc_median | -0.85497 |
| Bamboo | localdbShimmer | jitter_ddp | 0.792017 |
| Bamboo | localdbShimmer | jitter_local | 0.784232 |
| Bamboo | localdbShimmer | jitter_local_absolute | 0.744129 |
| Bamboo | localdbShimmer | jitter_ppq5 | 0.798735 |
| Bamboo | localdbShimmer | jitter_rap | 0.792017 |
| Bamboo | localdbShimmer | shimmer_apq11 | 0.920641 |
| Bamboo | localdbShimmer | shimmer_apq3 | 0.941375 |
| Bamboo | localdbShimmer | shimmer_apq5 | 0.95564 |
| Bamboo | localdbShimmer | shimmer_dda | 0.941375 |
| Bamboo | localdbShimmer | shimmer_local | 0.956345 |
| Bamboo | localdbShimmer | shimmer_local_db | 0.947616 |
| Bamboo | apq3Shimmer | hnr_ac_mean | -0.76251 |
| Bamboo | apq3Shimmer | hnr_ac_median | -0.76169 |
| Bamboo | apq3Shimmer | hnr_cc_mean | -0.78345 |
| Bamboo | apq3Shimmer | hnr_cc_median | -0.78636 |
| Bamboo | apq3Shimmer | jitter_ddp | 0.763633 |
| Bamboo | apq3Shimmer | jitter_local | 0.757236 |
| Bamboo | apq3Shimmer | jitter_local_absolute | 0.742558 |
| Bamboo | apq3Shimmer | jitter_ppq5 | 0.787746 |
| Bamboo | apq3Shimmer | jitter_rap | 0.763633 |
| Bamboo | apq3Shimmer | shimmer_apq11 | 0.854966 |
| Bamboo | apq3Shimmer | shimmer_apq3 | 0.965851 |
| Bamboo | apq3Shimmer | shimmer_apq5 | 0.932262 |
| Bamboo | apq3Shimmer | shimmer_dda | 0.965851 |
| Bamboo | apq3Shimmer | shimmer_local | 0.920444 |
| Bamboo | apq3Shimmer | shimmer_local_db | 0.883091 |
| Bamboo | aqpq5Shimmer | hnr_ac_mean | -0.76595 |
| Bamboo | aqpq5Shimmer | hnr_ac_median | -0.76605 |
| Bamboo | aqpq5Shimmer | hnr_cc_mean | -0.78921 |
| Bamboo | aqpq5Shimmer | hnr_cc_median | -0.79476 |
| Bamboo | aqpq5Shimmer | jitter_ddp | 0.727172 |
| Bamboo | aqpq5Shimmer | jitter_local | 0.737414 |
| Bamboo | aqpq5Shimmer | jitter_local_absolute | 0.746052 |
| Bamboo | aqpq5Shimmer | jitter_ppq5 | 0.766162 |
| Bamboo | aqpq5Shimmer | jitter_rap | 0.727172 |
| Bamboo | aqpq5Shimmer | shimmer_apq11 | 0.925337 |
| Bamboo | aqpq5Shimmer | shimmer_apq3 | 0.948538 |
| Bamboo | aqpq5Shimmer | shimmer_apq5 | 0.971221 |
| Bamboo | aqpq5Shimmer | shimmer_dda | 0.948538 |
| Bamboo | aqpq5Shimmer | shimmer_local | 0.947595 |
| Bamboo | aqpq5Shimmer | shimmer_local_db | 0.913301 |
| Bamboo | apq11Shimmer | hnr_ac_mean | -0.7598 |
| Bamboo | apq11Shimmer | hnr_ac_median | -0.76292 |
| Bamboo | apq11Shimmer | hnr_cc_mean | -0.77892 |
| Bamboo | apq11Shimmer | hnr_cc_median | -0.78588 |
| Bamboo | apq11Shimmer | jitter_local | 0.706313 |
| Bamboo | apq11Shimmer | jitter_local_absolute | 0.715148 |
| Bamboo | apq11Shimmer | jitter_ppq5 | 0.72881 |
| Bamboo | apq11Shimmer | shimmer_apq11 | 0.951959 |
| Bamboo | apq11Shimmer | shimmer_apq3 | 0.890628 |
| Bamboo | apq11Shimmer | shimmer_apq5 | 0.942785 |
| Bamboo | apq11Shimmer | shimmer_dda | 0.890628 |
| Bamboo | apq11Shimmer | shimmer_local | 0.929131 |
| Bamboo | apq11Shimmer | shimmer_local_db | 0.908408 |
| Bamboo | ddaShimmer | hnr_ac_mean | -0.76251 |
| Bamboo | ddaShimmer | hnr_ac_median | -0.76169 |
| Bamboo | ddaShimmer | hnr_cc_mean | -0.78345 |
| Bamboo | ddaShimmer | hnr_cc_median | -0.78636 |
| Bamboo | ddaShimmer | jitter_ddp | 0.763633 |
| Bamboo | ddaShimmer | jitter_local | 0.757236 |
| Bamboo | ddaShimmer | jitter_local_absolute | 0.742558 |
| Bamboo | ddaShimmer | jitter_ppq5 | 0.787746 |
| Bamboo | ddaShimmer | jitter_rap | 0.763633 |
| Bamboo | ddaShimmer | shimmer_apq11 | 0.854966 |
| Bamboo | ddaShimmer | shimmer_apq3 | 0.965851 |
| Bamboo | ddaShimmer | shimmer_apq5 | 0.932262 |
| Bamboo | ddaShimmer | shimmer_dda | 0.965851 |
| Bamboo | ddaShimmer | shimmer_local | 0.920444 |
| Bamboo | ddaShimmer | shimmer_local_db | 0.883091 |
| Bamboo | hnr | hnr_ac_max | 0.729795 |
| Bamboo | hnr | hnr_ac_mean | 0.956365 |
| Bamboo | hnr | hnr_ac_median | 0.957205 |
| Bamboo | hnr | hnr_cc_max | 0.734522 |
| Bamboo | hnr | hnr_cc_mean | 0.963114 |
| Bamboo | hnr | hnr_cc_median | 0.963653 |
| Bamboo | hnr | jitter_ddp | -0.77117 |
| Bamboo | hnr | jitter_local | -0.77831 |
| Bamboo | hnr | jitter_local_absolute | -0.83969 |
| Bamboo | hnr | jitter_ppq5 | -0.76828 |
| Bamboo | hnr | jitter_rap | -0.77117 |
| Bamboo | hnr | shimmer_apq11 | -0.81111 |
| Bamboo | hnr | shimmer_apq3 | -0.83483 |
| Bamboo | hnr | shimmer_apq5 | -0.83136 |
| Bamboo | hnr | shimmer_dda | -0.83483 |
| Bamboo | hnr | shimmer_local | -0.85989 |
| Bamboo | hnr | shimmer_local_db | -0.85463 |
| Bamboo | mean_pitch | fundamental_frequency_mean | 0.994381 |
| Bamboo | mean_pitch | fundamental_frequency_median | 0.991292 |
|  | | | |
| Phonation | Average Fundamental Frequency | fundamental_frequency_max | 0.705622 |
| Phonation | Average Fundamental Frequency | fundamental_frequency_mean | 0.896113 |
| Phonation | Average Fundamental Frequency | fundamental_frequency_median | 0.863356 |
| Phonation | Mean Fundamental Frequency | fundamental_frequency_mean | 0.837996 |
| Phonation | Mean Fundamental Frequency | fundamental_frequency_median | 0.865432 |
| Phonation | Mean Fundamental Frequency | jitter_local_absolute | -0.70852 |
| Phonation | Average Pitch Period | fundamental_frequency_mean | -0.8377 |
| Phonation | Average Pitch Period | fundamental_frequency_median | -0.86511 |
| Phonation | Average Pitch Period | jitter_local_absolute | 0.708635 |
| Phonation | Highest Fundamental Frequency | fundamental_frequency_max | 0.714716 |
| Phonation | Highest Fundamental Frequency | fundamental_frequency_mean | 0.727726 |
| Phonation | Lowest Fundamental Frequency | fundamental_frequency_mean | 0.734368 |
| Phonation | Lowest Fundamental Frequency | fundamental_frequency_median | 0.834336 |
| Phonation | Lowest Fundamental Frequency | jitter_local_absolute | -0.77582 |
| Phonation | Total Number Detected Pitch Periods | fundamental_frequency_mean | 0.798954 |
| Phonation | Total Number Detected Pitch Periods | fundamental_frequency_median | 0.826983 |
| Phonation | Total Number Detected Pitch Periods | jitter_local_absolute | -0.7705 |
| Phonation | Mean CPP F0 | fundamental_frequency_max | 0.717285 |
| Phonation | Mean CPP F0 | fundamental_frequency_mean | 0.827692 |
| Phonation | Mean CPP F0 | fundamental_frequency_median | 0.763739 |
|  | | | |
| DDK /ta/ | ddk_rate_ta | articulation_rate | 0.77018 |
| DDK /ta/ | ddk_rate_ta | avg_word_duration | -0.92673 |
| DDK /ta/ | ddk_rate_ta | speech_rate | 0.926727 |
| DDK /ta/ | ctv_ta | articulation_rate | -0.74632 |
| DDK /ta/ | ctv_ta | avg_word_duration | 0.941272 |
| DDK /ta/ | ctv_ta | speech_rate | -0.94127 |
